# Management of Hemodynamic and Respiratory Instability and Anesthetic Approaches in Patients Undergoing Pulmonary Thrombectomy for Pulmonary Embolism

**DOI:** 10.3390/jcm14082704

**Published:** 2025-04-15

**Authors:** Susana González-Suárez, John Camacho Oviedo, José Maria Suriñach Caralt, Maria Grao Roca, Isuru M. Dammala Liyanage, Mercedes Pérez Lafuente, Elisabeth Mena Muñoz, Carla González Junyent, María Martínez-Martínez, Daniel Barnés Navarro, Juan Carlos Ruíz-Rodríguez

**Affiliations:** 1 Department of Surgery, Universitat Autònoma de Barcelona, Unitat Docent Vall d’Hebron, Pg. de la Vall d’Hebron, 119-129, 08035 Barcelona, Spain; 2 Department of Anesthesiology, Hospital Universitari Vall d’Hebron, Pg. de la Vall d’Hebron, 119-129, 08035 Barcelona, Spain; 3 Cardiovascular Diseases Research Group, Vall d’Hebron Institut de Recerca (VHIR), Pg. de la Vall d’Hebron, 119-129, 08035 Barcelona, Spain; 4 Department of Radiology, Hospital Universitari Vall d’Hebron, Pg. de la Vall d’Hebron, 119-129, 08035 Barcelona, Spain; 5 Department of Medicine, Universitat Autònoma de Barcelona, Unitat Docent Vall d’Hebron, Pg. de la Vall d’Hebron, 119-129, 08035 Barcelona, Spain; 6 Department of Internal Medicine, Hospital Universitari Vall d’Hebron, Pg. de la Vall d’Hebron, 119-129, 08035 Barcelona, Spain; 7 Department of Intensive Care, Hospital Universitari Vall d’Hebron, Pg. de la Vall d’Hebron, 119-129, 08035 Barcelona, Spain; 8 Shock, Organ Dysfunction and Resuscitation (SODIR) Research Group, Vall d’Hebron Institut de Recerca (VHIR), Pg. de la Vall d’Hebron, 119-129, 08035 Barcelona, Spain

**Keywords:** pulmonary embolism, pulmonary mechanical thrombectomy, hemodynamic instability, hypotension, cardiorespiratory arrest

## Abstract

**Background/Objectives:** The incidence, timing, and predictors of hemodynamic and respiratory deterioration in patients with high-risk or intermediate-high-risk pulmonary embolism (PE) undergoing pulmonary mechanical thrombectomy (PMT) remain poorly understood. This hemodynamic and respiratory instability can lead to modifications in the anesthetic management. This study investigates these key factors and quantifies the 30-day mortality following thrombectomy. **Methods:** A retrospective study was conducted on 98 patients aged ≥18 years who underwent PMT. Patients were categorized based on the occurrence of cardiac arrest (CA). **Results:** Of the 98 patients, 34 had high-risk PE, 62 intermediate/high-risk, and 2 low risk. There were 27 cases of CA, 17 pre- and 10 intra-PMT. An SBP < 90 mmHg increases the risk of CA by 33 (*p* < 0.001); men have an 8-fold higher risk than women (*p* = 0.004); SpO_2_ <90% by 6 (*p* = 0.012); and pre-existing respiratory conditions increase the risk by 4 (*p* = 0.047)). N-terminal pro-B-type natriuretic peptide (NT-proBNP) levels were 8206 ± 11660.86 and 2388.50 ± 5683.71 pg/mL (*p* = 0.035) in patients with and without CA, respectively. During PMT, 14% of patients required increased vasoactive drug use, and 38.77% were intubated, including 12 who required ECMO support. Sedation was administered in 64.3% of patients, while general anesthesia was used in 38.8%, with a preemptive indication in 23.5%. The survival rate of patients without CA before and/or during PMT was 96%. **Conclusions:** While PMT was successfully performed in all patients, hemodynamic and respiratory instability remained a significant concern. More than 10% of patients experienced severe hemodynamic instability, primarily during thrombus extraction, requiring conversion from sedation to general anesthesia. Male sex, pre-existing respiratory disease, SpO_2_ < 90%, and SBP < 90 mmHg were associated with an increased risk of CA. Additionally, elevated NT-proBNP levels were linked to a higher incidence of CA.

## 1. Introduction

Pulmonary embolism (PE) affects approximately 1 in 1000 people worldwide each year and is the third leading cause of cardiovascular death [1,2]. Patients with intermediate-high-risk or high-risk PE, particularly those who are unresponsive to or have contraindications for systemic thrombolytic therapy, may benefit from pulmonary mechanical thrombectomy (PMT) [3]. Over the past decade, advancements in device technology have helped reduce hemorrhagic complications [4]. However, despite the lower bleeding risk associated with PMT, concerns regarding hemodynamic deterioration in these patients persist.

PE accounts for 2–9% of all out-of-hospital cardiac arrests (CA) and 5–6% of in-hospital CA [5,6]. These numbers may be underestimated due to frequent underdiagnosis of PE in clinical practice [2]. Some studies have found that up to 10% of CA initially attributed to primary cardiac causes were later identified as secondary to PE [7]. Large pulmonary vessel obstruction, combined with factors that increase pulmonary artery pressure—such as pain, anxiety, hypoxemia, hypercapnia, mechanical ventilation-induced airway pressure, and manipulation of the pulmonary arterial system—can lead to circulatory shock and, ultimately, CA [8].

Although complications and mortality in high- and intermediate/high-risk PE are documented [3], data on anesthetic techniques, hemodynamic and respiratory support strategies, and factors contributing to instability—especially during PMT—remain limited. Accordingly, this study examines these aspects with a focus on PMT.

## 2. Materials and Methods

### 2.1. Ethics Statement and Regulation

The Ethics Committee approved this single-center study at Vall d’Hebron University Hospital, Barcelona, Spain (PR(AG)102/202), dated 19 April 2024, and it was registered at www.clinicaltrials.gov on 22 April 2024 (registration number: NCT06525480). The requirement for informed consent was waived. The study included consecutive patients with PE who underwent PMT from 27 August 2014 to 23 August 2024.

### 2.2. Study Design and Participants

This retrospective observational study included all consecutive patients aged over 18 years with PE who underwent PMT (diagnosed by computed tomography angiography or lung perfusion scan). Exclusion criteria were patients under 18 years of age and those who did not undergo PMT.

### 2.3. Study Outcomes

Primary Objective: To determine the incidence, timing, and predictors of hemodynamic and respiratory deterioration in patients with PE undergoing PMT, as well as to describe the hemodynamic and respiratory support provided.

Secondary Objectives: To identify the different anesthetic techniques used in patients undergoing PMT and to assess overall mortality within the first 30 days post-PMT.

### 2.4. Data Collection and Data Measurement

Before the PMT procedure, the following data were recorded: anthropometric data, underlying conditions, PE risk factors, right ventricular dysfunction, biomarkers (N-terminal pro-B-type natriuretic peptide (NT-proBNP), cardiac troponins, D-dimers), need for vasoactive drugs, presence of acute severe hemodynamic instability (systolic blood pressure (SBP) < 60 mmHg) requiring bolus adrenaline, occurrence of CA, ECMO placement, and PE risk stratification [3] (Table 1).

During the procedure, the following data were recorded: airway devices (nasal cannula, ventimask^®^, oxygen mask with reservoir bag, optiflow™ nasal high flow therapy, orotracheal tube, ECMO); anesthesia procedures (none, sedation, sedation at the start of the procedure requiring conversion to general anesthesia during the procedure, continuation of general anesthesia started prior to PMT, and elective general anesthesia at the start of the procedure); administration of vasoactive drugs, occurrence of hemoptysis, type of catheter used for PMT, presence of acute severe hemodynamic instability (SBP < 60 mmHg) with bolus adrenaline requirement, and onset of respiratory and/or hemodynamic instability requiring intubation and/or ECMO support for CA. Thirty days post-PMT, mortality and its causes were recorded.

### 2.5. Data Analysis and Statistical Plan

A data analysis and statistical plan was developed after the data were accessed. For categorical variables, differences in parameters between the two groups (with CA/without CA) were assessed using the non-parametric Pearson’s chi-squared test. For continuous variables, differences in parameters between the two groups (with CA/without CA) were evaluated using the Mann–Whitney test (non-parametric), due to the non-normality of the variables confirmed by the Shapiro–Wilk test. The significance level used was 5% (α = 0.05). To identify potential risk factors for CA, a binary logistic regression (logit) was performed. The clinical risk factors considered included sex, age, SBP < 90 mmHg, HR ≥ 110 bpm, SpO_2_ < 90%, arterial hypertension, diabetes mellitus, cardiopathy, pulmonary pathology, renal pathology, cancer history, and pulmonary hypertension. The Hosmer–Lemeshow test assessed the goodness-of-fit of the predicted probabilities (*p*-value > 0.05). To minimize the impact of overfitting and multicollinearity, stepwise variable selection models were applied.

## 3. Results

The study included 98 patients (39.8% women). The mean age was 63.6 years (±13.5 years). Thirty-four patients had high-risk PE, 62 had intermediate/high-risk PE, and 2 patients had low-risk PE. Systemic fibrinolysis was administered prior to pulmonary thrombectomy in 11 patients with high-risk PE. During the thrombectomy procedure, 50% of the patients received an initial urokinase bolus directly into the thrombus (ranging from 200.000 to 400.000 IU).

### 3.1. Underlying Conditions and PE Etiology According to the Occurrence of CA

Seventeen (17.3%) patients experienced CA prior to PMT, and 10 (10.2%) experienced CA during PMT. Two patients who had CA during the procedure also had CA previously. Additionally, one patient before the procedure and three patients during PMT experienced adverse hemodynamic conditions with SBP < 60 mmHg, requiring adrenaline.

Preoperative factors associated with a higher frequency of CA were male sex (*p* = 0.019), SBP < 90 mmHg (*p* = 0.001), SpO_2_ < 90% (*p* = 0.040), and high-risk PE (*p* < 0.001) (Table 2). During PMT, 17.6% of patients with high-risk PE had CA, compared to 6.45% of those with intermediate/high-risk PE (*p* = 0.522).

**Figure 1:**
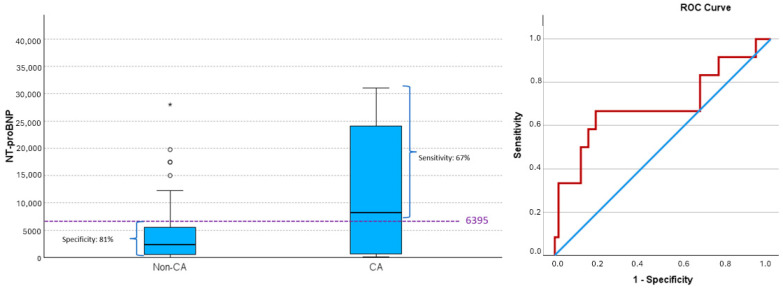
NT-proBNP values and risk of cardiac arrest (CA). Box plot by CA and ROC curve for predicting CA based on NT-proBNP values. Values of sensitivity and specificity, determined by the cut-off maximizing the Youden index (dashed line), are shown. An NT-proBNP value of ≥6395 achieves the most balanced rates of false negatives and false positives. This value closely aligns with the 75th percentile of NT-proBNP levels in the non-CA group. Above this threshold, more than 50% of NT-proBNP values in the CA group are found, while only 25% of NT-proBNP values in the non-CA group exceed this level Extreme outlier is indicated by asterisk (*), while moderate outliers are indicated by small open circles (∘). ROC curve; blue line: reference line, red line: NT-proBNP. CA, cardiac arrest; non-CA, non-cardiac arrest.

Elevated NT-proBNP levels were also associated with a higher frequency of CA (*p* = 0.035). NT-proBNP values ≥ 6395 led to CA in 67% of cases, while values < 6395 did not lead to CA in 81% of cases (positive predictive value (PPV) 42.1% and negative predictive value (NPV) 92.2%) (Figure 1) (AUC 0.694, 95% CI (0.499, 0.889), *p* = 0.049), with 6395 being the cutoff that maximized the Youden index).

**Figure 2:**
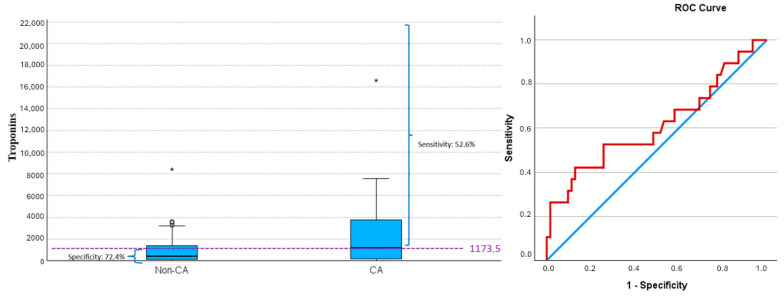
Troponin values and risk of cardiac arrest (CA). Box plot by CA and ROC curve for predicting CA based on troponin values. Values of sensitivity and specificity, determined by the cut-off maximizing the Youden index (dashed line), are shown. A troponin value of ≥1173.5 achieves the most balanced rates of false negatives and false positives. Extreme outliers are indicated by asterisk (*), while moderate outliers are indicated by small open circles (∘). ROC curve; blue line: reference line, red line: troponins. CA, cardiac arrest; non-CA, non-cardiac arrest.

Troponin values ≥1173.5 led to CA in 52.6% of cases, while values <1173.5 did not lead to CA in 74.2% of cases (PPV 38.5% and NPV 83.6%) (Figure 2) (AUC 0.615, 95% CI (0.455, 0.776), *p* = 0.159) with 1173.5 being the cutoff that maximized the Youden index).

**Figure 3:**
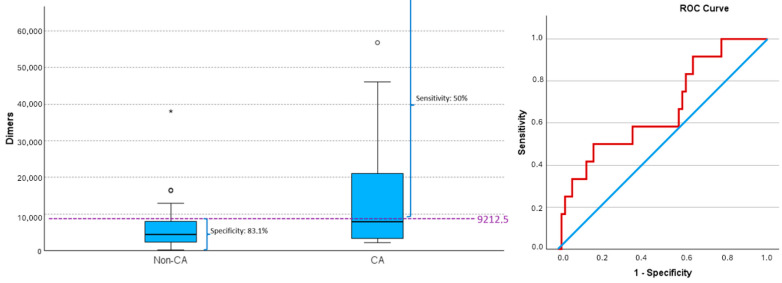
D-dimer values and risk of cardiac arrest (CA). Box plot by CA and ROC curve for predicting CA based on D-dimers values. Values of sensitivity and specificity, determined by the cut-off maximizing the Youden index (dashed line), are shown. A D-dimer value of ≥9212.5 achieves the most balanced rates of false negative and false positives. Extreme outlier is indicated by asterisk (*), while moderate outliers are indicated by small open circles (∘). CA, cardiac arrest; non-CA, non-cardiac arrest. ROC curve; blue line: reference line, red line: D-dimer.

D-dimer values ≥9212.5 led to CA in 50% of cases, while values <9212.5 did not lead to CA in 83.1% of cases (PPV 37.5% and NPV 89.1%) (Figure 3) (AUC 0.667, 95% CI (0.495, 0.839, *p* = 0.057), with 9212.5 being the cutoff that maximized the Youden index).

The clinical predictors of mortality incorporated into the final model, with the highest explanatory power, were sex, SBP < 90 mmHg, SpO_2_ < 90%, and the presence of pre-existing respiratory disease. The Hosmer–Lemeshow test, with a chi2 = 6.682, *p* = 0.463 (>0.05), indicated a good model fit. The AUC was excellent, with a value of 0.902, 95% CI [0.842–0.962]. Males had an 8-fold increased risk of CA compared to females (OR = 8.250, 95% CI [1.960–34.72], *p* = 0.004). SBP < 90 mmHg increased the risk of CA by 33 times (OR = 33.534, 95% CI [7.296–154.120], *p* < 0.001). SpO_2_ < 90% increased the risk of CA by 6 times (OR = 6.389, 95% CI [1.505–27.127], *p* = 0.012). The presence of pre-existing respiratory disease increased the risk of CA by 4 times (OR = 4.128, 95% CI [1.021–16.696], *p* = 0.047).

### 3.2. Hemodynamic and/or Respiratory Support

Before PMT, 27 patients (27.6%) received hemodynamic support with noradrenaline, with this administration being six times more frequent in those who experienced CA (*p* < 0.001). Dobutamine was used in 10 patients (10.20%), being fifteen times more frequent in CA cases (*p* < 0.001). During PMT, 38 patients (38.77%) received noradrenaline, with consumption 2.5 times higher in those with CA (*p* = 0.009). Dobutamine was administered to 13 patients (13.26%), with no significant differences based on the occurrence of CA (*p* = 0.447). Adrenaline was used in 29 patients: 25 with CA and 4 with SBP < 60 mmHg (1 before and 3 during PMT). In total, 14% of patients showed an increase in vasoactive drug consumption during the procedure (McNemar, *p* < 0.001).

Among the 13 patients with hemodynamic or respiratory deterioration during PMT (10 with CA and 3 with SBP < 60 mmHg), deterioration occurred after anesthetic induction and orotracheal intubation in 1 patient (8%), at the end of the procedure in 1 patient (8%), during urokinase administration in 2 (15%), during pulmonary arterial catheterization in 2 (15%), and during thrombectomy in 7 (53.8%). The Indigo^®^ and FlowTriever systems were used in 83 and 15 patients, respectively. No patient experienced severe hemodynamic instability with FlowTriever.

During PMT, hemodynamic instability occurred in 7 patients with high-risk PE (6 with CA and 1 with SBP < 60 mmHg) and in 6 patients with intermediate/high-risk PE (4 with CA and 2 with SBP < 60 mmHg).

In the context of CA, ECMO support was implemented in 9 patients before PMT and in 2 during the procedure. Additionally, one patient received ECMO support before PMT due to hemodynamic deterioration without CA. ECMO support lasted between 1 and 15 days (mean ± SD: 3.42 ± 3.90 days). The most used airway devices during the thrombectomy procedure were ventimask^®^, nasal glasses, and tracheal tube (Table 3).

### 3.3. Anesthesia During PMT

The different anesthetic procedures are shown in Table 3. Sedation was performed in 63% of patients, and general anesthesia in 38%. Significant differences were observed between the type of anesthesia and the PE risk (Table 4). Sedation was the most used anesthetic procedure in intermediate/high-risk patients compared to high-risk patients, while high-risk patients more frequently received general anesthesia as a continuation of prior anesthesia (*p* < 0.001). The incidence of general anesthesia was more than three times higher in high-risk PE patients than in those with intermediate/high-risk PE (*p* < 0.001).

The four patients who received elective general anesthesia for PMT (*n* = 4) included two with high-risk PE and two with intermediate/high-risk PE. Anesthetic induction was carried out with fentanyl, etomidate, and/or midazolam, and rocuronium, and two patients received propofol (0.5 mg/kg). All four patients survived, although one of them experienced CA after anesthetic induction and orotracheal intubation. Of the 10 patients who required conversion from sedation to general anesthesia during the procedure, 7 experienced severe hemodynamic instability, 2 had hemoptysis, and 1 had hypoxia.

### 3.4. Mortality

**Figure 4:**
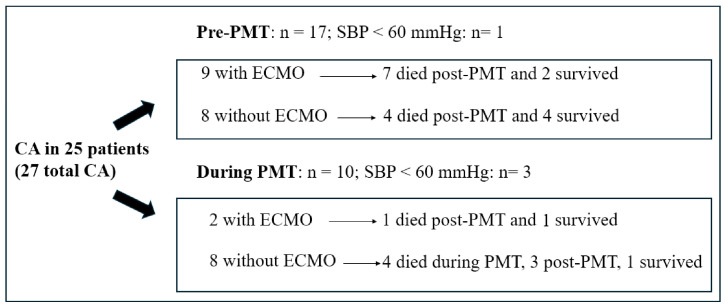
Mortality in patients with cardiac arrest pre-PMT or during PMT. CA, cardiac arrest; PMT, pulmonary mechanical thrombectomy.

The overall mortality rate was 22.44% (22 patients), including 4 deaths during PMT. Death occurred within the first six days after thrombectomy. Mortality was higher in high-risk patients (44%) compared to those with intermediate-high-risk PE (11.3%), particularly among those who experienced CA before or during PMT (18 patients). Of the 17 patients who experienced CA prior to the procedure, 6 survived, whereas only 2 of the 10 patients who had CA during PMT survived. Among patients who received ECMO support in the context of CA, three survived (Figure 4).

The primary factor compromising patient outcomes was cerebral ischemia, leading to brain death in nine cases. Other contributing factors included hemoptysis in two patients, mixed shock (cardiogenic and septic) in two patients, and cardiogenic shock in two others. Additionally, one patient developed aortic dissection, another experienced hemorrhagic shock, and one more suffered from multiorgan failure.

## 4. Discussion

This study underscores the frequent occurrence of hemodynamic deterioration in patients undergoing PMT for high-risk or intermediate-high-risk PE, particularly during thrombus extraction. Approximately 10% of these patients experienced severe cardiovascular instability—defined as CA or SBP <60 mmHg—prompting conversion to general anesthesia and increased use of vasopressors and inotropes in 14% of cases. This hemodynamic compromise was more common in high-risk PE (20.58%) but was also observed in intermediate-high-risk cases (9.67%). Moreover, mechanical ventilatory support was required in 38.77% of patients, including 12 who necessitated ECMO support. Male sex, pre-existing respiratory disease, SpO_2_ < 90%, and SBP < 90 mmHg were independently associated with an increased risk of CA. To date, specific predictive factors for CA in these patients have not been clearly identified. However, some authors [9,10] have observed that patients who experienced cardiovascular collapse or death during mechanical thrombectomy often presented with clinical features of high-risk pulmonary embolism (PE), such as severe hemodynamic compromise, right ventricular dysfunction, and delayed initiation of treatment. Other studies, although not focused exclusively on predicting CA, have suggested that biomarkers such as NT-proBNP, troponins, and D-dimer may serve as reliable indicators for assessing PE severity and prognosis, particularly NT-proBNP, in relation to right ventricular dysfunction, which can precede adverse cardiac events [11,12]. Our findings support that NT-proBNP provides superior predictive value for CA in this population, offering more clinically relevant information than troponins or D-dimer. The interpretation of these biomarkers should always be integrated with clinical findings and diagnostic results for a comprehensive risk assessment.

The severe hemodynamic instability observed in these patients influenced the choice of anesthetic technique. Notably, general anesthesia was predominantly employed in high-risk pulmonary embolism (PE) patients, whereas procedural sedation was more commonly used in those with intermediate-high-risk PE. PMT was performed exclusively under sedation in 73.8% of intermediate-high-risk patients; however, 16.2% of these patients experienced hemodynamic instability during the procedure. Similarly, while 23.5% of high-risk patients underwent PMT with sedation alone, 11.8% required conversion to general anesthesia intra-procedure. Sedation helps mitigate sympathetic stimulation and the subsequent increase in pulmonary artery pressures, but it must be carefully managed to prevent deep sedation, which can lead to hypoventilation, hypoxemia, and hypercapnia. Conversely, although general anesthesia should be avoided due to its potential to compromise hemodynamic stability, it remains the technique of choice in selected cases [13]. In this study, it was the second most used procedure (38.77%). A total of 23.5% of patients arrived intubated to the radiology room, and 15.30% required general anesthesia during PMT, primarily due to extreme hemodynamic or respiratory alterations. This technique should also be considered in cases of agitation, altered mental state, inability to maintain the supine position, pulmonary hemorrhage, hypoxemia, or risk of aspiration. No medication should be contraindicated as long as its deleterious effects are considered [14]; however, the risk of obstructive shock remains, as observed in one patient who experienced CA after intubation. In such cases, it would be advisable to have ECMO teams available, especially for high-risk patients, to manage cardiovascular instability post-intubation [15]. General anesthesia and positive pressure ventilation may precipitate right ventricular failure, compromise ventricular filling, and result in shock or CA [16,17,18,19]. Therefore, it is essential to have a single intravenous access for administering vasopressor and inotropic hemodynamic support prior to anesthetic induction [20,21]. Invasive blood pressure monitoring, accurate dosing of anesthetic drugs, and careful use of crystalloids via a second intravenous access are equally essential to minimize vasodilation, negative inotropic effects, and the increase in right ventricular end-diastolic pressure.

Given these hemodynamic challenges, optimizing respiratory support is equally critical. In most patients, oxygen administration through ventimask^®^ and nasal glasses (39.79%) was sufficient, although high-flow oxygen devices (8.16%) or a tracheal tube (38.77%) were also required in patients who were either already intubated or needed tracheal intubation during the procedure. The goal should be to maintain maximum inspiratory pressures <30 cmH_2_O, along with an individualized application of PEEP, low tidal volume ventilation (6 mL/kg of ideal body weight), and respiratory rates that ensure normocapnia [21,22].

Reported mortality rates for patients with high-risk and intermediate-high-risk PE are significant, estimated at approximately 30% and 2–15%, respectively [23,24,25,26,27]. Published mortality rates for patients undergoing PMT [28,29,30] range from 2% to 4.6%, which contrasts with the findings of our study. However, these discrepancies are likely attributable to differences in patient inclusion criteria. Notably, 17.34% of patients in our cohort experienced CA prior to thrombectomy, a critical hemodynamic condition not documented in these previous studies [28,29,30]. Given the limited number of patients who received ECMO, particularly in the context of CA, it is not possible to draw definitive conclusions regarding its effect on survival. However, its benefits are well-documented, including ventricular decompression, improved systemic perfusion, and a reduction in vasopressor therapy and myocardial oxygen demand [31,32,33,34]. It is possible that earlier initiation of ECMO support in high-risk patients who have not yet experienced CA may be associated with a more favorable prognosis by preventing the negative consequences of inadequate organ perfusion linked to CA. In this context, low systolic blood pressure (SBP), low SpO_2_ levels, and elevated NT-proBNP levels [35] could serve as early warning signs of an increased risk of CA and the need for ECMO initiation. As observed in our study, it would be valuable to further investigate and confirm whether elevated NT-proBNP levels represent a predictive parameter for CA risk in patients with pulmonary embolism. In some studies, the use of extracorporeal circulation devices has proven useful in patients with PE who experienced CA [36,37,38]. A systematic review, which included case reports of massive PE, found that 51.2% of patients received ECMO support after CA, and of these, 51.2% survived [39]. Additionally, Giraud et al. [40] also reported good results using veno-venous (VV) and veno-arterial (VA) ECMO in two patients. However, most available studies are limited to case reports, which introduces considerable bias into the results [39,40,41]. During PMT, the role of ECMO support in the context of CA has been observed by some authors [9]. In a retrospective single-center study of 151 patients with intermediate to high risk, Benfor et al. [42] recorded the occurrence of CA in nine patients (6%), four of whom received successful ECMO, while the other five died intraoperatively. The 30-day overall mortality they observed was 8%, with no deaths occurring in patients rescued with ECMO. In any case, the role of ECMO support in patients who experience CA in the context of PE remains unclear. A multicentric, randomized controlled trial would be needed to determine the best treatment. Moreover, the multiple complications associated with this therapy must be considered, which increase morbidity and mortality as the duration of the therapy extends [42,43].

Regarding the impact of catheter type on the occurrence of hemodynamic/respiratory instability during TMP, we did not observe any complications with the FlowTriever system. Currently, most research focuses on the general efficacy and safety of these devices without providing details on instability episodes during the procedure. A retrospective study of 27 high-risk PE patients treated with the FlowTriever system reported a significant reduction in mean pulmonary arterial pressure and heart rate after the procedure, with no major device-related complications [44]. Similarly, a review of the Indigo^®^ aspiration system highlighted its effectiveness in pulmonary artery recanalization and its safety profile in intermediate-risk PE patients [45]. Therefore, further research is needed to determine if one system induces more hemodynamic/respiratory instability than the other during the procedure.

Our study has several limitations. Its retrospective nature prevented the accurate recording of all anesthetic drugs administered, as well as the exact doses of vasoactive and inotropic medications used during PMT. Additionally, key data such as pulmonary artery pressure values were not collected for all patients. Lastly, the low percentage of patients who received ECMO support did not allow for the evaluation of whether this therapy offers significant advantages in the context of CA.

## 5. Conclusions

In summary, although PMT was successfully performed in all patients, concerns persist regarding hemodynamic and/or respiratory decompensation during the procedure. More than 10% of patients may experience severe hemodynamic deterioration during PMT. Hemodynamic and respiratory support, as well as the choice of anesthetic technique, are primarily influenced by the severity of the PE. Male sex, pre-existing respiratory disease, SpO_2_ < 90%, and SBP < 90 mmHg were associated with an increased risk of cardiac arrest, along with elevated NT-proBNP levels, which could serve as early indicators for the onset of cardiac arrest.

## Figures and Tables

**Table 1:** Risk stratification for pulmonary embolism.

Early Mortality Risk		Indicators of Risk			
		Shock or Hypotension *	PESI Class III-V or sPESI ≥ 1 **	Signs of RV Dysfunction on an Imaging Test	Cardiac Laboratory Biomarkers
High		+	+	+	+
Intermediate	Intermediate high	−	+	+	+
	Intermediate low	−	+	Either 1 (or none) positive	
Low		−	−	Assessment optional: if assessed, both negative	

PESI, Pulmonary Embolism Severity Index; sPESI, simplified Pulmonary Embolism Severity Index; RV: right ventricular, * Definition of the European Society of Cardiology (ESC) (3). One of the following: need for cardiopulmonary resuscitation, systolic blood pressure (SBP) <90 mmHg or vasopressors required to achieve an SBP ≥ 90 mm Hg despite adequate filling status and end-organ hypoperfusion (altered mental status, cold, clammy skin, oliguria/anuria, increased serum lactate), systolic BP <90 mmHg or systolic BP drop ≥40 mmHg, lasting longer than 15 min and not caused by new-onset arrhythmia, hypovolemia or sepsis. ** sPESI: Age > 80 years, history of cancer, history of previous lung or cardiovascular disease, heart rate (HR) greater than or equal to 110 beats per minute, systolic blood pressure less than 100 mmHg, oxygen saturation less than 90%. One of the variables present is considered high risk.

**Table 2:** Baseline parameters. Previous diseases, etiology of pulmonary embolism, hemodynamic and biochemical parameters. Values are number (proportion) or (number) mean ± SD.

Pre-Thrombectomy Parameters	CA			*p*-Value
	Total	Non-CA	CA	
	*n* = 98	*n* = 73	*n* = 25	
Hypertension	56 (57.1)	39 (53.4)	17 (68)	*p* = 0.20
Diabetes mellitus	15 (15.3)	11 (15.1)	4 (16)	*p* = 0.91
Cardiopathy	17 (17.3)	12 (16.4)	5 (20)	*p* = 0.68
Respiratory diseases	26 (26.5)	17 (23.3)	7 (28)	*p* = 0.21
Renal failure	14 (14.3)	10 (13.7)	4 (16)	*p* = 0.77
Cancer history	22 (22.4)	16 (21.9)	6 (24)	*p* = 0.82
Pulmonary hypertesion	41 (41.8)	33 (45.2)	8 (32)	*p* = 0.24
Current cancer	18 (18.4)	11 (15.1)	7 (28)	*p* = 0.18
Undetermined cause of PE	40 (40.8)	31 (42.4)	9 (36)	
COVID-19	10 (10.2)	6 (8.2)	4 (16)	
Obesity-sedentary lifestyle	8 (8.2)	7 (9.6)	1 (4)	
Morbid obesity	3 (3.1)	3 (4.1)	0 (0)	
Traumatism-fractures	7 (7.1)	7 (9.6)	0 (0)	
Postoperative	6 (6.1)	3 (4.1)	3 (12)	
Contraceptives	2 (2)	2 (2.7)	0 (0)	
Coagulation disorders	2 (2)	2 (2.7)	0 (0)	
Ruptured hydatid cyst	1 (1)	0 (0)	1 (4)	
Single ventricle	1 (1)	1 (1.4)	0 (0)	
PE severity:				*p* < 0.001
High	34 (35.1)	15 (20.8)	19 (76)	
Intermediate/high	62 (63.2)	57 (78)	5 (20)	
Low	2 (2.1)	1 (1.4)	1 (4)	
SBP < 90 mmHg	35 (35.7)	16 (21.9)	19 (76)	*p* < 0.001
Heart rate > 110 bpm	33 (33.7)	27 (37)	6 (24)	*p* = 0.23
Oxygen saturation < 90%	21 (21.4)	12 (16.4)	9 (36)	*p* = 0.04
Bilateral PE	91 (94.8)	68 (95.8)	23 (92)	*p* = 0.46
Unilateral PE	4 (4.1)	2 (2.8)	2 (8)	*p* = 0.25
NT-proBNP	(70) 5467.44 ± 7548.07	(58) 4142.34 ± 5683.71	(12) 11,872.08 ± 11,660.86	*p* = 0.03
Troponins	(81) 1890.17 ± 5628.99	(62) 968.37 ± 1394.48	(19) 4898.16 ± 11,034.37	*p* = 0.13
D-dimers	(71) 8772.96 ± 14,747.68	(59) 7397 ± 13,680.47	(12) 15,536 ± 18,362.53	*p* = 0.07
SPS (mmHg)	(33) 46.70 ± 14.06	(26) 47.69 ±14.60	(13) 43 ± 12.11	*p* = 0.47

Number (proportion) are shown for categorical variables. (Number) mean ± SD are shown for continuous variables. *p*-values from chi^2^ test or Mann–Whitney test are shown. CA, cardiac arrest; PE, pulmonary embolism; SBP, systolic blood pressure; NT-proBNP, N-terminal pro-B-type natriuretic peptide; SPS, systolic pulmonary pressure.

**Table 3:** Anesthetic procedures, airway devices and hemodynamic instability during thrombectomy. Values are number (proportion).

Anesthesia	*n* = 98 (100%)	Hemodynamic Instability (*n* = 13)	Airway Devices	*n* = 98 (100%)
Sedation	53 (54.1)	None	Ventimask^®^ Nasal glasses Oxygen mask with reservoir bag Optiflow^TM^ nasal high flow therapy Oxygen mask with reservoir bag + ECMO	19 (19.38) 20 (20.4) 8 (8.16) 5 (5.10) 1 (1)
Continuation of general anesthesia initiated prior to PMT	23 (23.5)	3 CA (1 ECMO) 1 SBP < 60 mmHg	Orotracheal tube (before procedure) OTT + ECMO (both before procedure) OTT before procedure + ECMO during procedure	13 (13.26) 9 (9.2) 1 (1)
Sedation at the start, with conversion to general anesthesia during PMT	10 (10.2)	5 CA 2 SBP < 60 mmHg	Oxygen mask with reservoir bag at the start + OTT during procedure Ventimask^®^ at the start + OTT during procedure Nasal glasses at the start + OTT during procedure Optiflow^TM^ nasal high flow therapy at the start + OTT during	4 (4.08) 3 (3.1) 2 (2) 1 (1)
None	7 (7.1)	None	Oxygen mask with reservoir bag Optiflow^TM^ nasal high flow therapy Ventimask^®^ at the start + oxygen mask with reservoir bag during Ventimask^®^	1 (1) 2 (2) 1 (1) 3 (3.1)
Elective general anesthesia at the beginning of the PMT	4 (4.1)	1 CA (1 ECMO)	OTT at the start OTT at the start + ECMO during	3 (3.1) 1 (1)
Start without anesthesia, followed by general anaesthesia during PMT	1 (1)	1 CA	Ventimask^®^ at the start + OTT during procedure	1 (1)

CA: Cardiorespiratory arrest, SBP: Systolic blood pressure, PMT, pulmonary mechanical thrombectomy; OTT: orotracheal tube.

**Table 4:** Anesthetic procedures and risk stratification for pulmonary embolism. Values are number (proportion).

Anesthesia	Risk of PE			
	Total	Low	Intermediate/High	High
Total	98 (100)	2 (100)	62 (100)	34 (100)
Sedation	53 (54.6)	0 (0)	45 (73.8)	8 (23.5)
Continuation of general anesthesia initiated prior to PMT	23 (23.7)	2 (100)	2 (3.22)	19 (55.9)
Sedation at the start, with conversion to general anesthesia during PMT	10 (10.2)	0 (0)	8 (12.9)	2 (5.9)
None	7 (7.2)	0 (0)	4 (6.45)	3 (8.8)
Elective general anesthesia at the beginning of the PMT	4 (4.1)	0 (0)	2 (3.22) *	2 (5.9)
Start without anesthesia, followed by general anesthesia during PMT	1 (1)	0 (0)	1 (1.61)	0 (0)

PE, pulmonary embolism; PMT, pulmonary mechanical thrombectomy. * One patient with hemodynamic instability at the beginning of PMT.

## Data Availability

Data are contained within the article. The original contributions presented in this study are included in the article.

## Abbreviations

PMT	Pulmonary mechanical thrombectomy
CA	Cardiac arrest
NTproBNP	N-terminal pro-B-type natriuretic peptide
PE	Pulmonary embolism
SBP	Systolic blood pressure
PESI	Pulmonary Embolism Severity Index
sPESI	simplified Pulmonary Embolism Severity Index
RV	Right ventricle
OTT	Orotracheal tube
